# Extracts of select endemic plants from the Republic of Mauritius exhibiting anti-cancer and immunomodulatory properties

**DOI:** 10.1038/s41598-021-83461-0

**Published:** 2021-02-19

**Authors:** Shahin Kauroo, Joyce Govinden-Soulange, V. Mala Ranghoo-Sanmukhiya, Kathryn Miranda, William E. Cotham, Michael D. Walla, Mitzi Nagarkatti, Prakash Nagarkatti

**Affiliations:** 1 Faculty of Agriculture, University of Mauritius, Reduit, Moka Republic of Mauritius; 2 Department of Pathology, Microbiology & Immunology, School of Medicine, University of South Carolina, 202 Osborne Administration Building, Columbia, SC 29208 USA; 3 Department of Chemistry and Biochemistry, College of Arts and Science, University of South Carolina, Columbia, SC USA

**Keywords:** Drug discovery, Immunology, Plant sciences, Molecular medicine, Oncology

## Abstract

Mauritius Island possesses unique plant biodiversity with a potential reservoir of biologically active compounds of pharmacological interest. In the current study, we investigated Mauritius endemic plant families Asteraceae, Ebenaceae, Sapotaceae, and Erythroxylaceae, for anti-cancer properties on T cell lymphoma and B16F10 Melanoma cells and immunomodulatory properties on primary T and B cells. The cytotoxicity of methanolic plant extracts at 1, 10, 25 µg/ml was determined. The most active plant species were evaluated for their apoptosis-inducing effects. The immunomodulatory properties of the plants were also studied, and preliminary phytochemical screening of selected plants was done by LC–MS analysis. *Psiadia lithospermifolia* (Lam.) Cordem (Asteraceae) at 25 µg/ml was the most cytotoxic on both EL4 and B16 cells and triggered apoptosis by the death receptor pathway, and at least in part, by the mitochondrial pathway. Most plant species from Asteraceae, Ebenaceae, Erythroxylaceae, and Sapotaceae inhibited the proliferation of activated T and B cells, although some promoted T cell proliferation. LC–MS profile of Asteraceae plants showed the presence of terpenes, terpenoids, fatty acids, and phenolic. Flavonoids and phenolic acid were also detected from Ebenaceae and Sapotaceae plants. Together, our study demonstrated that Mauritius endemic flora exhibit potential anti-cancer and anti-inflammatory properties worthy of further in-depth studies.

## Introduction

**Figure 1:**
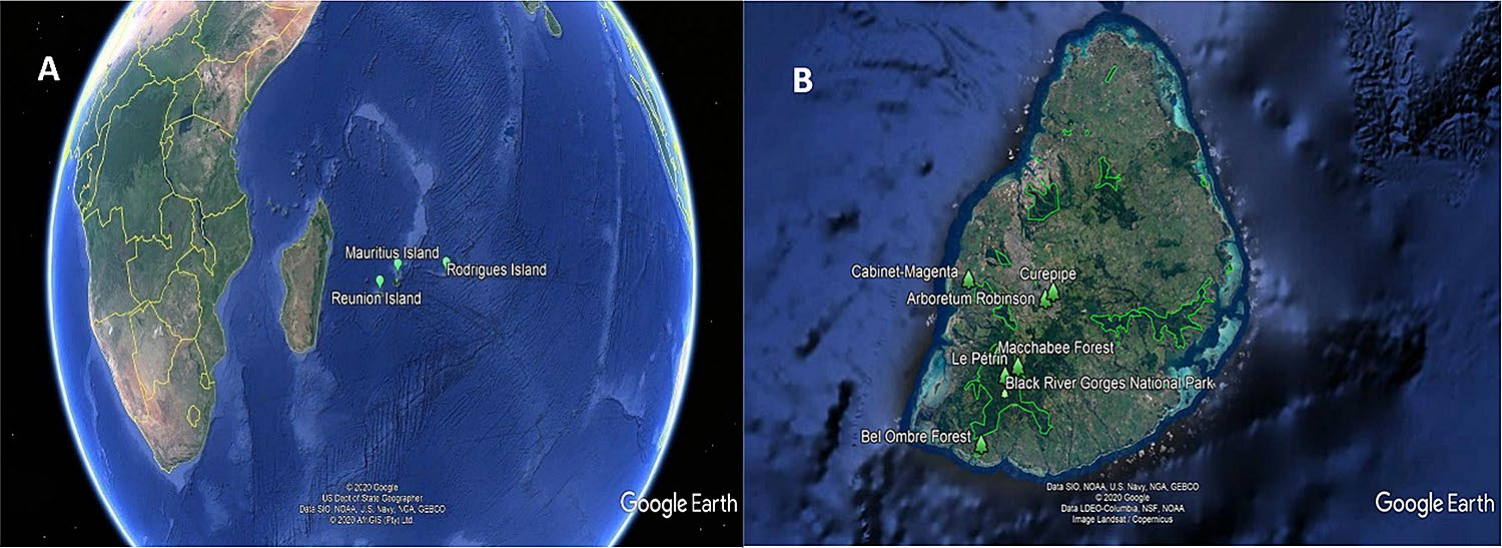
(**A**) Location of Mauritius and the Mascarenes Islands in the Indian Ocean. (**B**) Collection site of endemic plants across the island of Mauritius.

The Mascarene Islands include three volcanic islands named Réunion, Mauritius, and Rodrigues found in the east of Madagascar situated in a line along a submerged ridge found in the southeast coast of Africa in the Indian Ocean (Fig. 1A). The flora of the Mascarene Islands has a relatively high level of endemism because of the island’s geographical isolation. Mauritius Island is renowned for the extraordinary richness of its endemic flora, which is indicative of phytochemical diversity and is home to as many as 691 indigenous plant species of which 273 are single Island endemics.

Plant families namely Asteraceae, Ebenaceae, Erythroxylaceae, and Sapotaceae which are targeted in this study are well reported in local practice of ethnomedicine. The genus Psiadia Jacq. ex. Willd which belongs to the Asteraceae family is used in traditional medicine against various ailments, including treatment of abdominal pains, colds, fevers, bronchitis, and asthma^1^. As elaborated in a recent publication^2^, the leaf has the greatest healing properties, commonly used as leaf decoctions or as plaster for immobilizing fractures. Moreover, the Asteraceae species are famously known as a source of sesquiterpene lactones which are reported as promising apoptosis-inducing agents^3^. In Mauritius, the genus Diospyros L is the sole representative of the family Ebenaceae; the plants are used to treat infections caused by bacteria and fungi, against helminthic infections and malaria^4^. The Ebenaceae is commonly known as a prolific source of naphthoquinones such as isodiospyrin, which is a 1, 4-naphthoquinone that is highly cytotoxic isolated from the genus Diospyros^5^. Similarly, the family Erythroxylaceae is represented solely by the genus Erythroxylum P. Browne, whose species are renowned for the treatment of kidney stones^6^. Erythroxylaceae is characterized by the presence of tropane alkaloids such as pervilleines A, which is isolated from *Erythroxylum pervillei* that has been shown to exhibit promising activity as multidrug resistance (MDR) inhibitor^7^. This study deals with two genera from the Sapotaceae family namely Mimusops L and Sideroxylon L. These plants are used by local people to cure dysentery and diarrhea and to treat skin infections^8^. The triterpenoid saponins are commonly present in Sapotaceae and these compounds have been reported to possess potent cytotoxic activity^9^.

Cancer in Mauritius accounts for 12.8% of deaths recorded in 2016; the most common types of cancer among males is that of the prostate gland (14.2%), lungs (12.6%), and colorectum (12.5%). The main types of cancer among females are that of the breast (40.8%) followed by the uterus (12.7%). Cancer incidence is a big health challenge worldwide and the cancer burden in African countries is projected to rise to over two million cases by 2040. Despite the availability of chemotherapy, successful treatment of cancer remains a big challenge because of the various associated side effects, such as nonselective toxicity against normal cells, and drug resistance. Inflammation is defined as a response to stimulation by infections caused by invading pathogens or endogenous signals such as cell injury and is triggered by innate immunity followed by activation of the adaptive immunity. Inflammatory responses activate T and B lymphocytes resulting in the activation of macrophages, eosinophils, and ultimately secretion of antibodies, activation of complement cascade that plays a critical role in controlling infections and provide a defense to the host. Despite the sophistication of the immune system, when overactivated or not properly terminated, the immune system can cause substantial damage to the host leading to several inflammatory disorders^10^. Moreover, there is now clear evidence to demonstrate that chronic inflammation is also the underlying cause of several types of cancers. Thus, diet and nutrition that suppress chronic inflammation may help prevent the development of such cancers.

In the current study, the anti-cancer and anti-inflammatory properties of several plant extracts from Mauritius were therefore investigated with the goal that such studies would identify compounds that can be used to prevent and treat cancers. The extracts of plant species demonstrating promising tumoricidal activity such as *Psiadia lithospermifolia* (APL) were selected for further mechanistic study. The current study sheds light on the mode of action of APL through the induction of apoptosis in cancer cells via both the death receptor and mitochondrial pathways in EL4 T cell lymphoma (EL4) and B16F10 (B16) Melanoma cells. The anti-inflammatory and immunomodulatory activity of these extracts on concanavalin A (ConA) activated T cells and LPS-activated B cells from C57BL/6 mice were further investigated. Preliminary LC–MS analysis was also undertaken to identify major phytoconstituents that can explain the therapeutic potential of studied endemic plants.

## Material and methods

### Collection of plant material

Leaf samples of endemic plant species (Table 1) were collected from different parts of Mauritius Island (Fig. 1B). Voucher specimens were deposited at the Mauritius Herbarium (Ministry of Agro-Industry and Food Security) for accession number/Taxonomic number. The plant’s name in Table 1 has been checked on The Plant List website.

**Table 1:** Mauritius endemic plant species.

Family	Plant species	Accession no	Site of collection
Asteraceae	*Psiadia arguta* Voigt (APA)	MAU 26407	Arboretum Robinson Curepipe (National Park)
	*Psiadia lithospermifolia* Cordem (APL)	MAU 26404	
	*Psiadia viscosa* (Lam.) A.J. Scott (APV)	MAU 26406	
Ebenaceae	*Diospyros boutoniana* A.DC *(EDB)*	MAU 26412	Macchabee Forest (Black River Gorges National Park)
	*Diospyros nodosa* Poir *(EDN)*	MAU 26411	
	*Diospyros egrettarum* I. Richardson *(EDE)*	MAU 26513	Curepipe
	*Diospyros leucomelas* Poir *(EDL)*	MAU 26548	Black River Gorges National Park
	*Diospyros chrysophyllos* Poir *(EDC)*	MAU 26547	Bel Ombre Forest
	*Diospyros pterocalyx* Bojer ex A.DC *(EDP)*	MAU 26413	Le Pétrin (Black River Gorges National Park)
Erythroxylaceae	*Erythroxylum hypericifolium* Lam *(EEH)*	MAU 26548	Bel Ombre Forest
	*Erythroxylum laurifolium* Lam *(EEL)*	MAU 25561	Cabinet-Magenta Black River
Sapotaceae	*Mimusops balata* (Aubl.) C.F.Gaertn *(SMB)*	MAU 26408	Arboretum Robinson Curepipe (National Park)
	*Sideroxylon cinereum* Lam *(SSC)*	MAU 26409	
	*Sideroxylon puberulum* A.DC *(SSP)*	MAU 26410	
	*Sideroxylon boutonianum* A.DC *(SSB)*	MAU 23250	National park
	*Sideroxylon sessiliflorum* (Poir.) Capuron ex Aubrév *(SSS)*	MAU 2,251	National park

*Plant sample site location are represented in Fig. 1.

### Preparation of plant extracts

The collected mature leaf specimens were washed with distilled water and freeze-dried before extraction. Freeze-dried leaves were then ground into a fine powder and stored in air sealed black plastic bags at − 20 °C. The ground powders were extracted using the maceration method. The powder (150 g) was soaked in 450 ml cold methanol in a closed flask with intermittent shaking for 24 h. The mixture was filtered, and the residue was further extracted twice by using the same fresh solvent and all the filtrates were pooled together. Finally, from the filtrate, the solvent was evaporated under reduced pressure and low temperature. The resulting dry crude leaf extracts were stored in dark containers at 4 °C. The dry crude leaf extract was further resuspended in DMSO to obtain a working concentration of 1, 10, and 25 µg/ml of dry crude leaf extract.

### Mice

C57BL/6 female mice were purchased from the Jackson Laboratory (Bar Harbor, ME). All mice were housed in specific pathogen-free conditions at the AAALAC-accredited animal facility at the University of South Carolina, School of Medicine (Columbia, SC). All animal experiments were carried out in accordance with the National Institutes of Health guidelines and all protocols were approved by the University of South Carolina Institutional Care and Use Committee (IACUC).

### Cell lines

Murine cancer cell lines (EL-4 lymphoma (EL4) and B16F10 Melanoma (B16) cells, were maintained in complete RPMI medium supplemented with 10% heat-inactivated fetal bovine serum, 10 mM l-glutamine, 10 mM HEPES, and 100 µg/ml penicillin/streptomycin at 37 °C and 5% CO2, as described in^11,12^. These cell lines had been originally purchased from the American Type Culture Collection (ATCC), Manassas, Virginia. EL-4 cells grow as non-adherent cells while B16F10 cells grow as adherent cells in tissue culture flasks. Thus, when confluent, EL-4 cells were harvested directly while the B16F10 cells were harvested with trypsin/EDTA, washed three times with the RPMI 1640 medium, and then used in the assays as described previously^11,12^.

### Reagents

RPMI 1640 (Roswell Park Memorial Institute medium), l-glutamine, HEPES, Gentamicin, Phosphate buffer saline (PBS), and fetal bovine serum were purchased from Invitrogen (Carlsbad, CA). Concanavalin A (Con A), Methanol, DMSO, and 3,3′-Dihexyloxacarbocyanine Iodide (DIOC6) were purchased from Sigma-Aldrich (St-Louis, MO). TUNEL kits were obtained from Roche (Indianapolis, IN). Alexa Fluor 488 Annexin V/Dead cell Apoptosis Kits were procured from Invitrogen by Thermo Fisher Scientific. Lipopolysaccharide (LPS) was purchased from Invitrogen. Inhibitors against Caspase 8 (Z-IETD-FMK) and Caspase 9 (Z-LEHD-FMK) were purchased from R&D Systems (Minneapolis, MN). Plant extracts suspended in DMSO were used in the in vitro studies and DMSO was used as vehicle control.

### In vitro cytotoxic activity of Mauritius endemic plant extracts on tumor cells

B16 cells and EL4 cells were harvested and washed in RPMI medium and were plated in 96 well tissue culture plates which were then incubated at 37 °C for 24 h using 5% CO2 and 95% air. The cells were treated with different concentrations of plant extracts including 1, 10, and 25 µg/ml of dry crude leaf extract. The stock solution was diluted in culture medium ensuring that the DMSO concentration did not exceed 0.1%, a dose at which the cell viability is not affected. Control cells were incubated in culture medium with DMSO as vehicle. The plant extracts were tested in triplicate for their effect on cell viability.

### MTT assay

We used the MTT assay to check for the viability of cells. In this assay, cells reduce the yellow dye 3-(4,5-dimethyl-2-thiazolyl)-2,5-diphenyl-2H-tetrazolium bromide (MTT) to a blue formazan product. After incubation for 24 h, MTT solution was used to replace the medium as per company protocol. The plates were incubated for an additional 4 h after which the MTT reagent was removed and the formazan crystals were dissolved through the addition of 100% DMSO and gentle shaking. Reading was taken from Victor plate reader at 540 nm.

### Annexin V/propidium iodide (PI) apoptosis detection in EL4 and B16 cells treated with plant extracts

This assay was performed as described in our previous studies^13^. The cells were labeled as described by the manufacturer’s protocol provided by Alexa Fluor488 Annexin V/PI Cell Apoptosis Kit (Thermo Fisher Scientific Inc., Germany). Cells were treated with 1, 10, 25 μg/ml of dry crude leaf extract for 24 h. After treatment, the cells were rinsed twice with PBS at 4 °C. Next, we added 100 μl of annexin binding buffer to the cells suspended at a concentration of 1 × 10^6^ cells per ml followed by incubation with 5 μl Annexin-V of conjugated with Alexa488- and 5 μl of Propidium Iodide (PI) for 15 min in the dark at room temperature. Next, the cells were treated with the binding buffer followed by their analysis using flow cytometry.

### TUNEL apoptosis detection in EL4 and B16 cells treated with plant extracts

The TUNEL assay to detect apoptosis was performed as described previously^12,13^. Briefly, freshly cultured tumor cells were treated with vehicle (DMSO) or different concentrations such as 1, 10, 25 µg/ml of dry crude leaf extract for 24 h. Apoptosis in treated cancer cell lines was determined by performing TUNEL assays (FITC-dUTP nick-end labeling) using an in-situ Cell-Death Detection kit (Roche, Indianapolis, IN).

### Role of caspases in apoptosis induction of EL4 and B16 cells

To investigate the role of caspases in APL-induced Apoptosis in EL4 and B16 cells, we performed in vitro assays as described^12^. Both EL4 and B16 cells were incubated with caspases 8 and 9 inhibitors (20 µM) for at least 1 h before APL treatment. The cells were harvested after 24 h of treatment and TUNEL assays were performed to determine apoptosis as described earlier.

### Analysis of mitochondrial membrane potential (MMP)

Mitochondrial membrane potential (MMP) in EL4 and B16 cells after vehicle (DMSO) or APL treatment was determined using 3,3′-Dihexyloxacarbocyanine Iodide (DiOC6) dye as described previously^11,12^. At least three independent experiments were performed, and the data shown represent one of the experiments.

### ^3^H-Thymidine Uptake Assay for detection of Con A-induced proliferation of T cells and LPS-induced activation of B cells

Spleens were harvested from C57BL/6 mice. The cells (5 × 10^5^) were stimulated with Con A (2 μg/ml) or with LPS (100 μg/ml) plated in 96 well round bottom plates, along with different concentration of (1, 10, 25 μg/ml dry crude leaf extract or vehicle (DMSO) for 24 h, after which the cells were harvested and 2 μC_i_ (20 µl) of ^3^H-Thymidine was added. The radioactivity was recorded using a liquid scintillation counter (MicroBetaTrilux; PerkinElmer Life and Analytical Sciences).

### LC–MS analysis

#### Low mass resolution LC–MS in positive ion electrospray mode

We used the following mass spectrometer: Waters QT of API US, quadrupole time-of-flight instrument. The LC was a Dionex Ultimate 3000 UPLC. LC Column: Chromegabond WR C18, 2.1 mm × 150 mm, 3 μm particles (ES Industries). Injection volume: 5 μl. Binary gradient. Solvent A: water with 0.1% Formic acid. Solvent B: acetonitrile with 0.1% formic acid. Flow rate 200 μl/min. Initial conditions 5% B for 2 min, ramp to 95% B over 30 min. Hold at 95% B for 10 min.

#### Low mass resolution LC-MS in negative ion electrospray mode

We used the following mass spectrometer: Waters Premier XE triple quadrupole instrument. The LC was a Waters Acquity UPLC system. LC Column: Chromegabond WR C18, 2.1 mm × 150 mm, 3 μl particles (ES Industries). Injection volume: 8 μl Binary gradient. Solvent A: water with 0.1% Formic acid. Solvent B: acetonitrile with 0.1% formic acid. The flow rate of 200 μl/min. Initial conditions 5% B for 2 min, ramp to 95% B over 30 min. Hold at 95% B for 10 min.

#### High mass resolution LC–MS in positive and negative electrospray mode

We used the following mass spectrometer: Thermo Velos Pro Orbitrap. The LC was a Dionex U0gtltmate 3000 RSLCNano system. LC Column: Acclaim PepMap RSLC C18, 0.3 mm × 150 mm, 2 μm particles (Thermo Scientific). Binary gradient. Solvent A: water with 0.1% Formic acid. Solvent B: acetonitrile with 0.1% formic acid. Flow rate of 8 μl/min. Initial conditions 5% B for 2 min, ramp to 20%B over 2 min; ramp to 95%B over 21 min; Hold at 95% B for 15 min.

### Statistical analysis

Results presented here represent at least three independent experiments and are depicted as the Mean ± SEM. In some flow cytometry data presentation, a representative experiment has been depicted. Statistical analyses were performed using one-way ANOVA (Dunnett’s test) in GraphPad Prism as appropriate, with a *p*-value of ≤ 0.05 considered to be statistically significant. Histograms and dot plot diagrams were designed with the software FlowJo Version 9 for flow cytometry analysis. LC–MS analysis was performed on X Calibur and MasslynxV4.1.

## Results

### Cytotoxic effect of Mauritius endemic plant extracts against EL4 and B16 tumor cells

**Figure 2:**
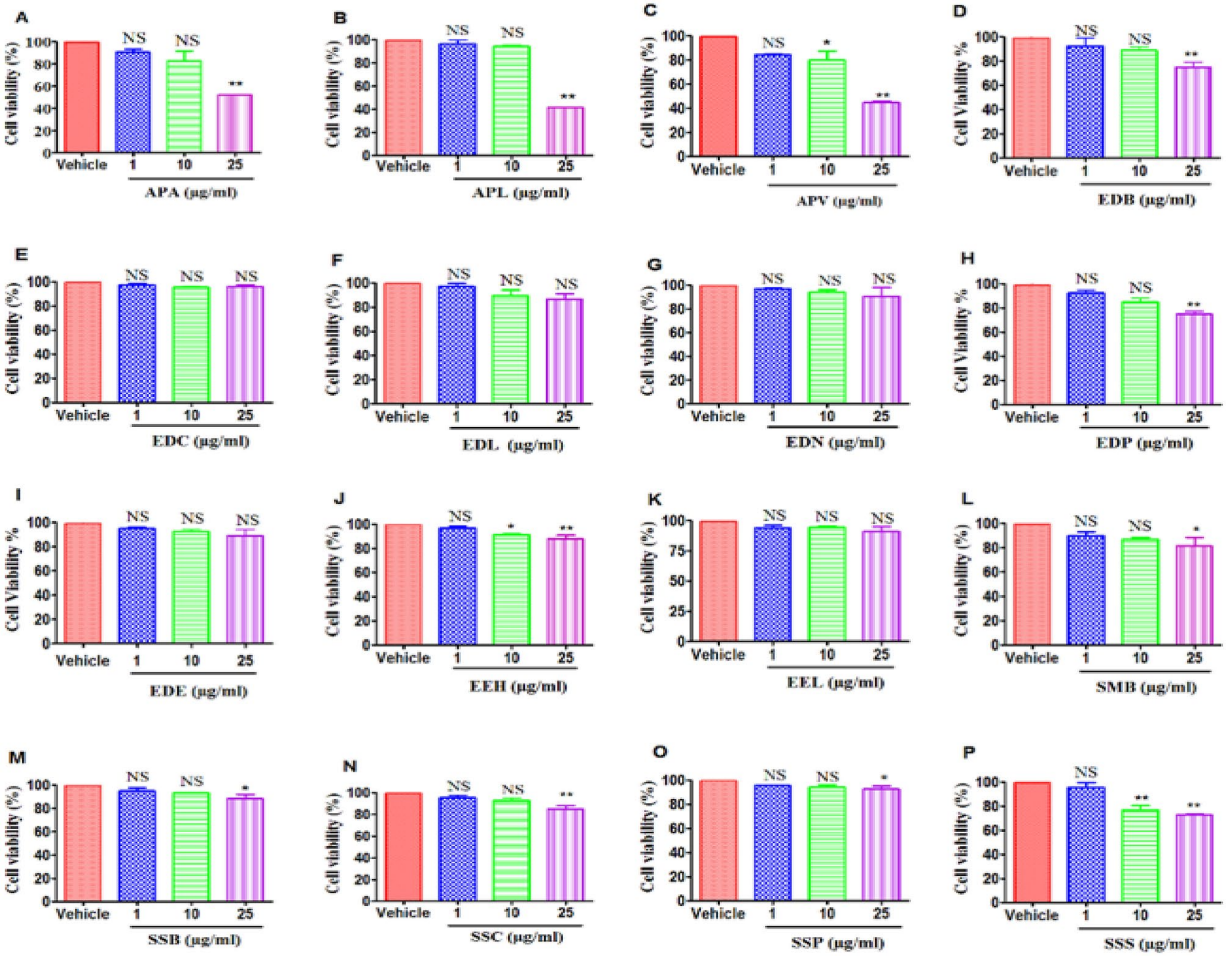
Effect of treatment of EL-4 cells with plant extracts on cell viability: EL-4 cells were cultured with various plant extracts or DMSO as vehicle, for 24 h. The cell viability was next measured by using the MTT test. (**A**–**C**) represent the Asteraceae family ((**A**) APA, (**B**) APL, (**C**) APV). (**D**–**I**) represent the Ebenaceae family ((**D**) EDB, (**E**) EDC, (**F**) EDL, (**G**) EDN, (**H**) EDP, (**I**) EDE). (**J**–**K**) represent the Erythroxylaceae family (J: EEH, K: EEL). L-P represent the Sapotaceae family ((**L**) SMB, (**M**) SSB, (**N**) SSC, (**O**) SSP, (**P**) SSS). The vertical bars represent mean ± S.E.M. of three independent experiments. The significance between various groups was tested using one-way ANOVA where NS: Not significant; (*)p < 0.05, (**)p < 0.01, (***)p < 0.001.

**Figure 3:**
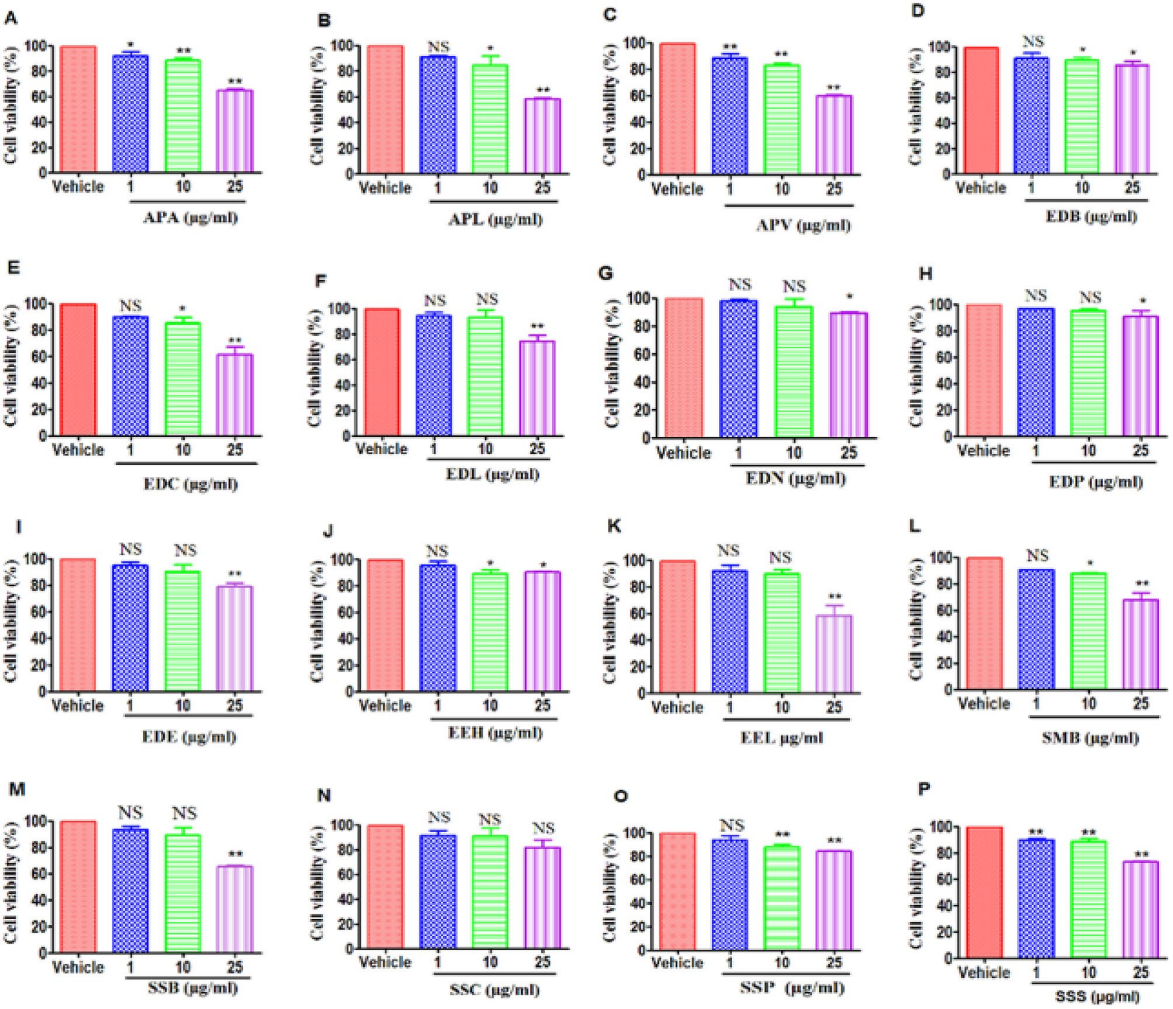
Effect of treatment of B16 melanoma cells with plant extracts on cell viability: EL-4 cells were cultured with various plant extracts or DMSO as vehicle, for 24 h. The cell viability was next measured by using the MTT test. (**A**–**C**) represent the Asteraceae family ((**A**) APA, (**B**) APL, (**C**) APV). (**D**–**I**) represent the Ebenaceae family ((**D**) EDB, (**E**) EDC, (**F**) EDL, (**G**) EDN, (**H**) EDP, (**I**) EDE). (**J**–**K**) represent the Erythroxylaceae family ((**J**) EEH, (**K**) EEL). (**L**–**P**) represent the Sapotaceae family ((**L**) SMB, (**M**) SSB, (**N**) SSC, (**O**) SSP, (**P**) SSS). The vertical bars represent mean ± S.E.M. of three independent experiments. The significance between various groups was tested using one-way ANOVA where NS: not significant; (*)p < 0.05, (**)p < 0.01, (***)p < 0.001.

To test for the anti-cancer cytotoxic effects of various Mauritius endemic plant methanol extracts, we used two types of tumor cells: EL-4 and B16 cells. Both cell lines were treated with vehicle (DMSO) or 1, 10, or 25 µg/ml of dry crude leaf extract in vitro for 24 h, and the viability was determined by performing an MTT assay. The results are graphically represented in Figs. 2 and 3. Our data demonstrated that treatment with the plant extracts APL (*Psiadia lithospermifolia*) (Figs. 2B, 3B), APA (*Psiadia arguta*) (Figs. 2A, 3A) and APV (*Psiadia viscosa*) (Figs. 2C, 3C) from the family Asteraceae at 25 μg/ml of dry crude leaf extract showed a potent decrease in the viability of both EL4 and B16 cells. APL showed 41.99 ± 0.37% (Fig. 2B) decreased in cell viability of EL4 cells. On the other hand, APL showed 56.61 ± 1.99% (Fig. 3B) decreased in cell viability of B16 melanoma cells. Treatment of B16 melanoma cells with EDC (Fig. 3E), EDL (Fig. 3F) and EDB (Fig. 3D) extract from Ebenaceae, EEH (Fig. 3J) and EEL (Fig. 3K) from Erythroxylaceae, SMB (Fig. 3L), SSB (Fig. 3M), SSC (Fig. 3N) and SSS (Fig. 3P) from Sapotaceae at 25 μg/ml caused a decrease in cancer cell viability. In summary, melanoma cells were more sensitive to killing by the majority of plant extracts at the highest doses tested when compared to lymphoma cells. Because APL showed promising cytotoxic activity, we used APL to performed additional studies.

### APL plant extracts induce apoptosis in EL4 and B16 tumor cells

**Figure 4:**
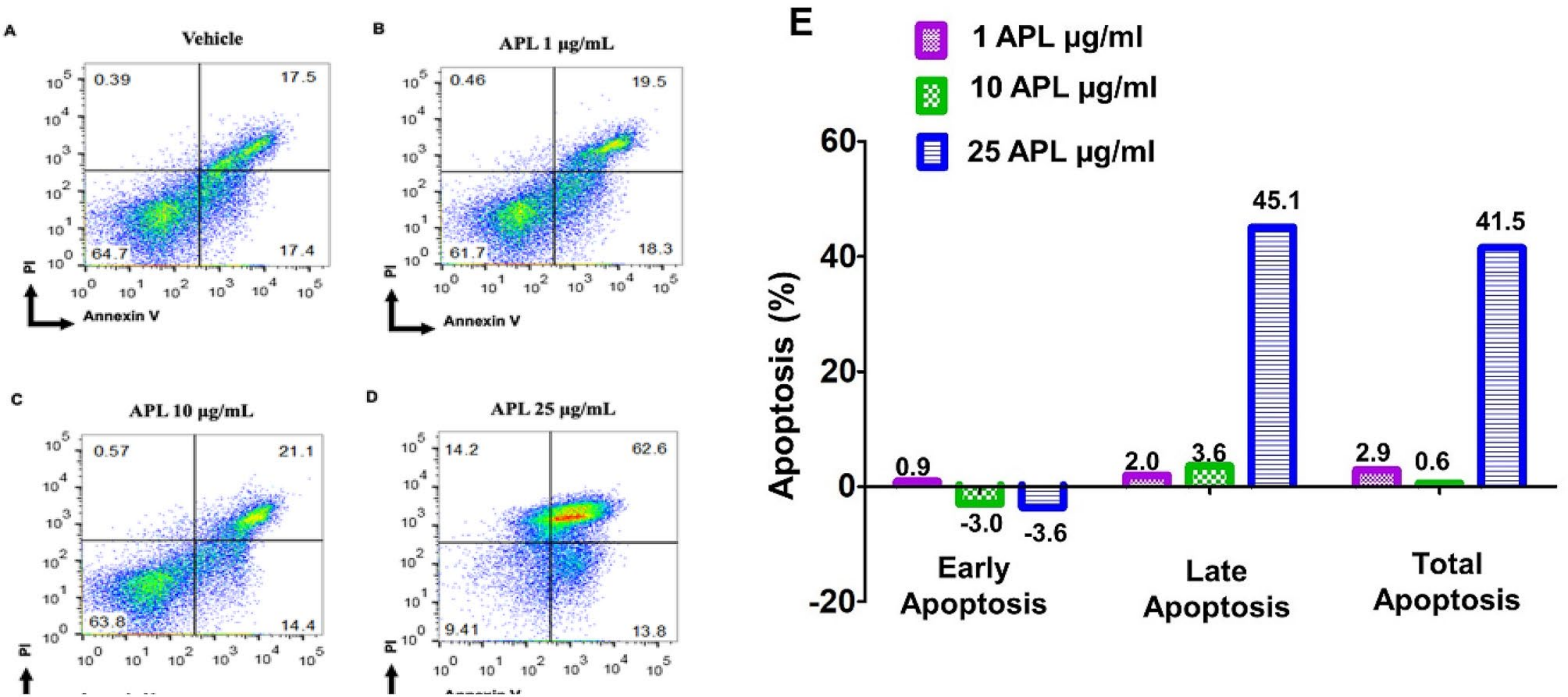
Apoptosis in EL4 cells by APL: This was determined by Annexin V-PI staining after 24 h treatment with vehicle (**A**) or APL (*Psiadia lithospermifolia*) at different concentrations such as 1 µg/ml (**B**), 10 µg/ml (**C**), 25 µg/ml (**D**). The data presented are representative of three independent experiments. Bar chart (**E**) shows quantitative data of average of three independent flow cytometry experiments with apoptosis in APL groups relative to vehicle controls.

**Figure 5:**
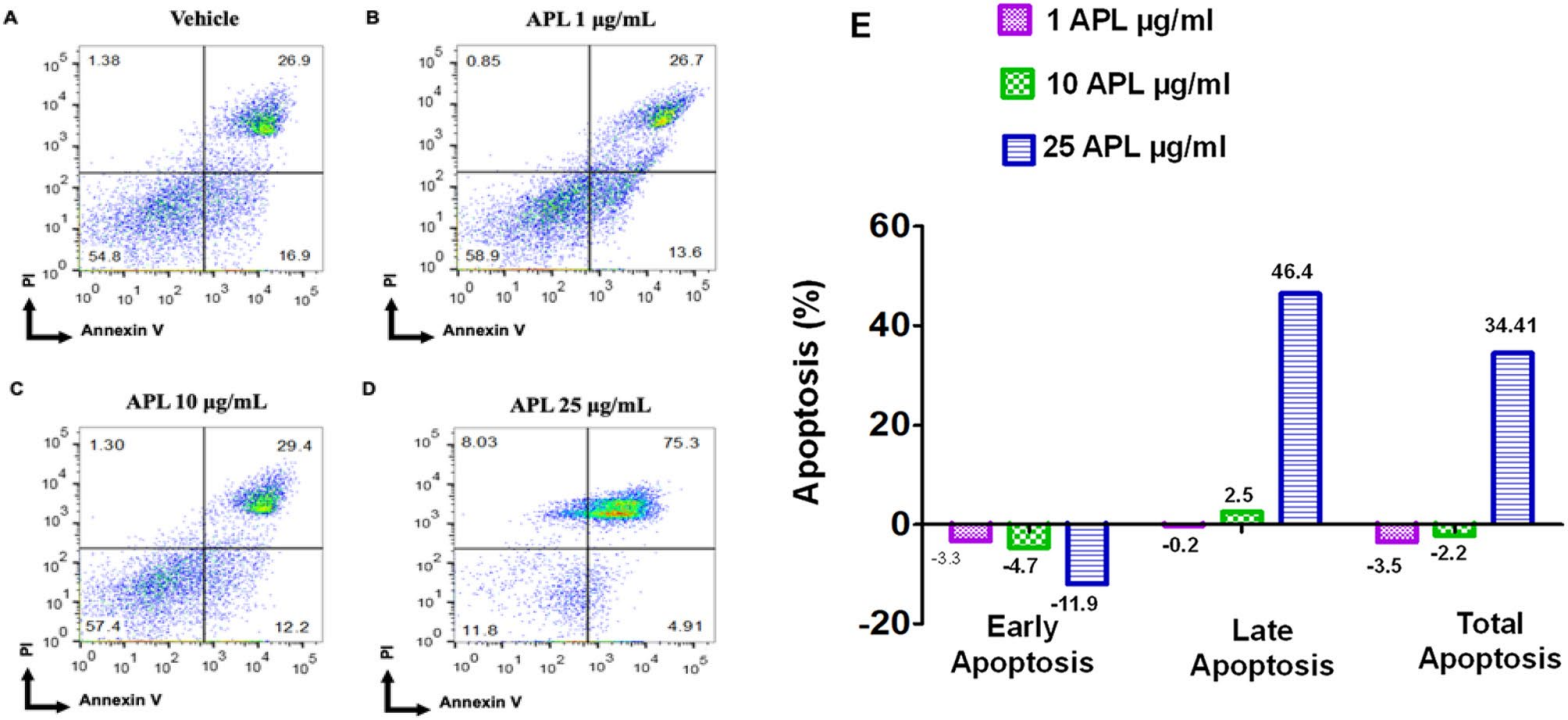
Apoptosis in B16 melanoma cells by APL: This was determined by Annexin V-PI staining after 24 h treatment with vehicle (**A**) or APL (*Psiadia lithospermifolia*) at different concentrations such as 1 µg/ml (**B**), 10 µg/ml (**C**), 25 µg/ml (**D**). The data presented are representative of three independent experiments. Bar chart (**E**) shows quantitative data of average of three independent flow cytometry experiments with apoptosis in APL groups relative to vehicle controls.

To understand if APL extracts were inducing apoptosis, we performed staining of cells with AnnexinV and PI. To this end, both cancer cell lines were treated with various doses of APL (1, 10, 25 µg/ml dry crude leaf extract) or DMSO for 24 h followed by staining of cells with Annexin V/PI followed by flow cytometric analysis. In this assay, cells stained for Annexin V only were indicative of early apoptotic cells and those stained for both Annexin V and PI are considered to be late apoptotic cells, while cells stained only for PI were considered to be necrotic cells. The data obtained from Annexin V/PI is graphically presented in a dot plot diagram in Figs. 4 and 5 which demonstrated that both EL4 and B16 cells underwent significant levels of early and or late apoptosis and necrosis in a dose-dependent manner. Treatment of EL4 cells with APL induced a dose-dependent increase in early apoptotic cells. At the highest dose tested (25 µg/ml of dry crude leaf extract), there was also an increase in late apoptosis (62.6%) and slowly shifting to necrotic cells (14.2%) (Fig. 4D). On the other hand, treatment of B16 melanoma cells at 25 μg/ml dose of APL caused a dramatic increase in late apoptotic cells (75.3%) shifting to necrotic cells (8.03%) (Fig. 5D). Because there was some background apoptosis in vehicle controls, the data were also expressed to indicate the level of apoptosis in extracts after subtracting the apoptosis seen in vehicle controls (Figs. 4E, 5E).

### APL plant extract triggers death-receptor pathway of apoptosis in EL4 and B16 cells

**Figure 6:**
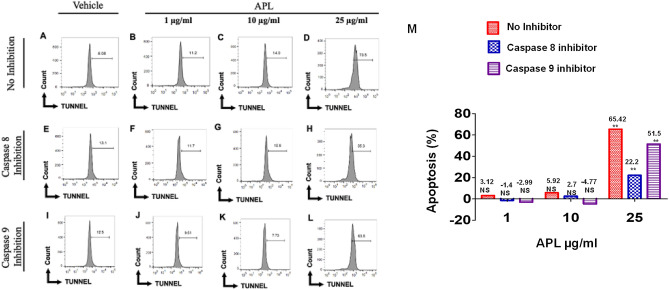
Role of caspase 8 and 9 in apoptosis induction in EL4 cells: This was determined by TUNNEL assay. Cells were treated at different concentrations with APL (*Psiadia lithospermifolia*) and vehicle or in the presence of inhibitors of caspase 8 and 9. (**A**–**D**) represent treatment without any inhibitors of caspases where (**A**) (vehicle DMSO), (**B**) (1 µg/ml), (**C**) (10 µg/ml), (**D**) (25 µg/ml) of APL. (**E**–**H**) Represent treatment with APL in presence of caspase 8 inhibitor. (**E**) (vehicle DMSO), (**F**) (1 µg/ml), (**G**) (10 µg/ml), (**H**) (25 µg/ml) of APL extract. (**I**–**L**) represent treatment with APL in presence of caspase 9 inhibitor. (**I**) (vehicle DMSO), (**J**) (1 µg/ml), (**K**) (10 µg/ml), (**L**) (25 µg/ml) of APL extract. The data presented are representative of three independent experiments. Bar chart (**M**) shows quantitative data of average of two independent flow cytometry experiments in which the values were expressed relative to no inhibitor. The significance between various groups was tested using one-way ANOVA where NS: not significant; (*)p < 0.05, (**)p < 0.01.

**Figure 7:**
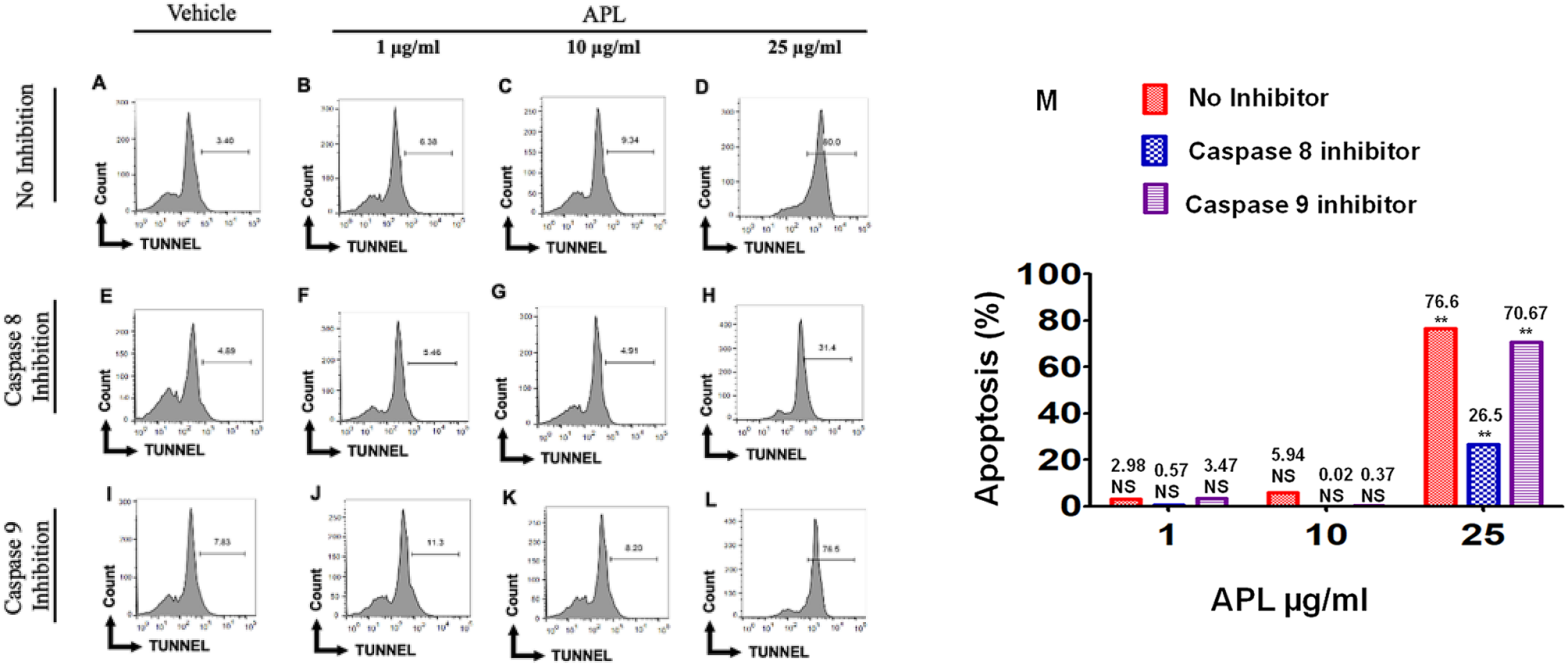
Role of caspase 8 and 9 in apoptosis induction of B16 melanoma cells: This was determined by TUNNEL assay. Cells were treated at different concentrations with APL (*Psiadia lithospermifolia*) and vehicle or in the presence of inhibitors of caspase 8 and 9. (**A**–**D**) represent treatment without any inhibitors of caspases where (**A**) (vehicle DMSO), (**B**) (1 µg/ml), (**C**) (10 µg/ml), (**D**) (25 µg/ml) of APL. (**E**–**H**) represent treatment with APL in presence of caspase 8 inhibitor. (**E**) (vehicle DMSO), (**F**) (1 µg/ml), (**G**) (10 µg/ml), (**H**) (25 µg/ml) of APL extract. (**I**–**L**) represent treatment with APL in presence of caspase 9 inhibitor. (**I**) (vehicle DMSO), (**J**) (1 µg/ml), (**K**) (10 µg/ml), (**L**) (25 µg/ml) of APL extract. The data presented are representative of three independent experiments. Bar chart (M) shows quantitative data of average of two independent flow cytometry experiments in which the values were expressed relative to no inhibitor. The significance between various groups was tested using one-way ANOVA where NS: Not significant; (*)p < 0.05, (**)p < 0.01.

To investigate the role of caspases in APL-induced apoptosis in EL4 and B16 cells, we performed caspase-blocking studies using caspase 8 inhibitor (Z-IETD-FMK) and Caspase 9 inhibitor (Z-LEHD-FMK), followed by TUNEL assay. Results are represented in the form of a histogram plot in Figs. 6 and 7. In the absence of caspase inhibitors, APL significantly induced apoptosis in EL4 (73.5%) (Fig. 6D) and B16 cells (80%) (Fig. 7D). However, the data demonstrated significant blocking of APL-induced apoptosis in EL4 cells in the presence of caspase-8 inhibitor (35.3%) (Fig. 6H) and to a lesser extent by caspase-9 inhibitor (63.8%) (Fig. 6L). Similarly, in B16 cells, caspase-8 inhibitor was more effective in blocking apoptosis (31.4%) (Fig. 7H) when compared to caspase-9 inhibitor (78.5%) (Fig. 7L). Cumulative data from multiple experiments with statistical analyses have been shown in Figs. 6M, 7M). These data suggested that APL-induced apoptosis was primarily mediated by death receptor (extrinsic) pathway and to a lesser extent through intrinsic pathways.

### APL plant extracts trigger intrinsic pathway of apoptosis in EL4 and B16 cells

**Figure 8:**
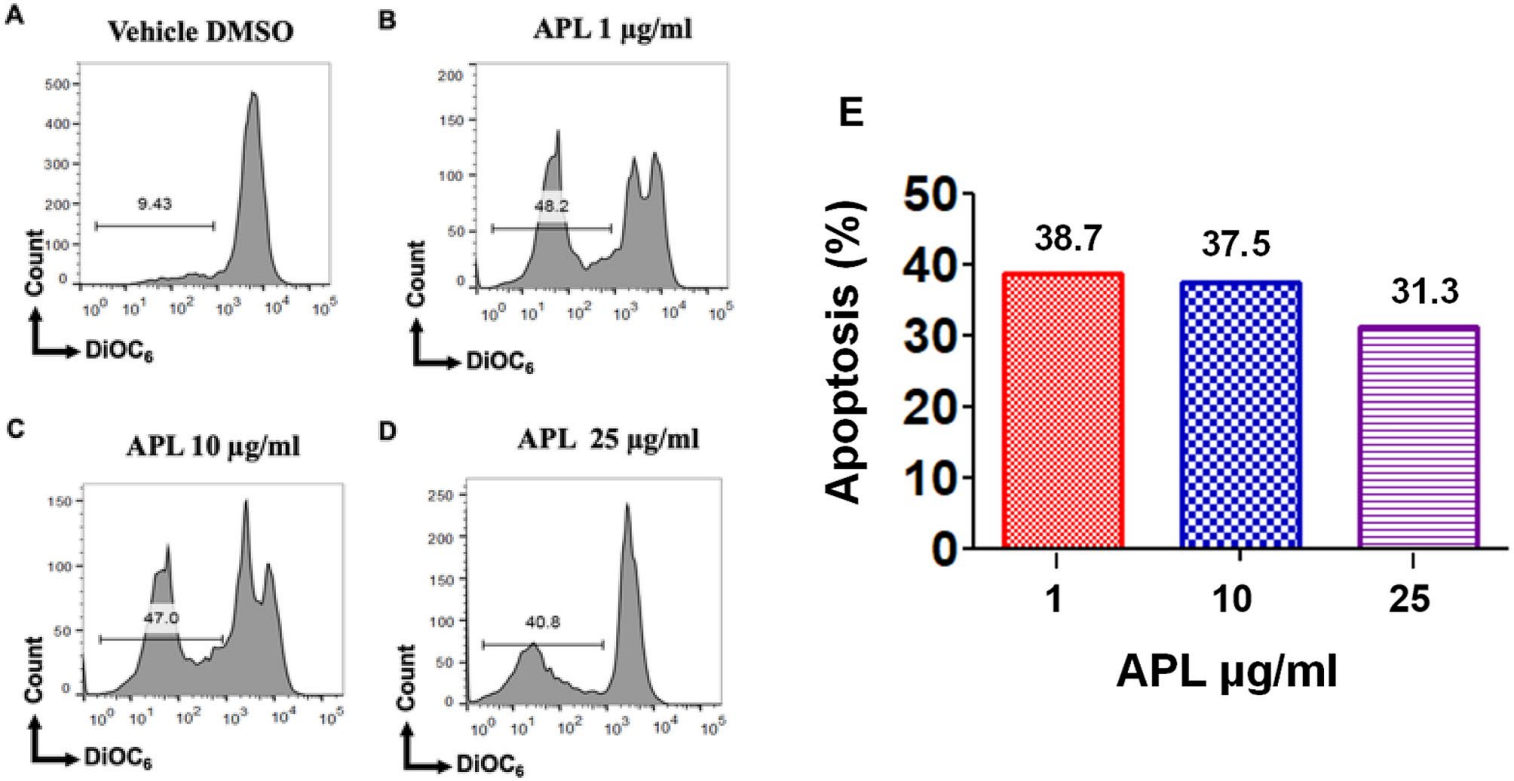
Effect of APL treatment on mitochondrial membrane potential (MMP) in EL4 cells: This was determined using 3,3′-Dihexyloxacarbocyanine Iodide (DiOC_6_) after treatment with APL (*Psiadia lithospermifolia*) or vehicle (DMSO). (**A**) (vehicle DMSO), (**B**) (1 µg/ml), (**C**) (10 µg/ml), (**D**) (25 µg/ml) of APL extract. The data presented are representative of three independent experiments. (**E**) shows data as mean % apoptosis relative to vehicle controls from two independent experiments.

**Figure 9:**
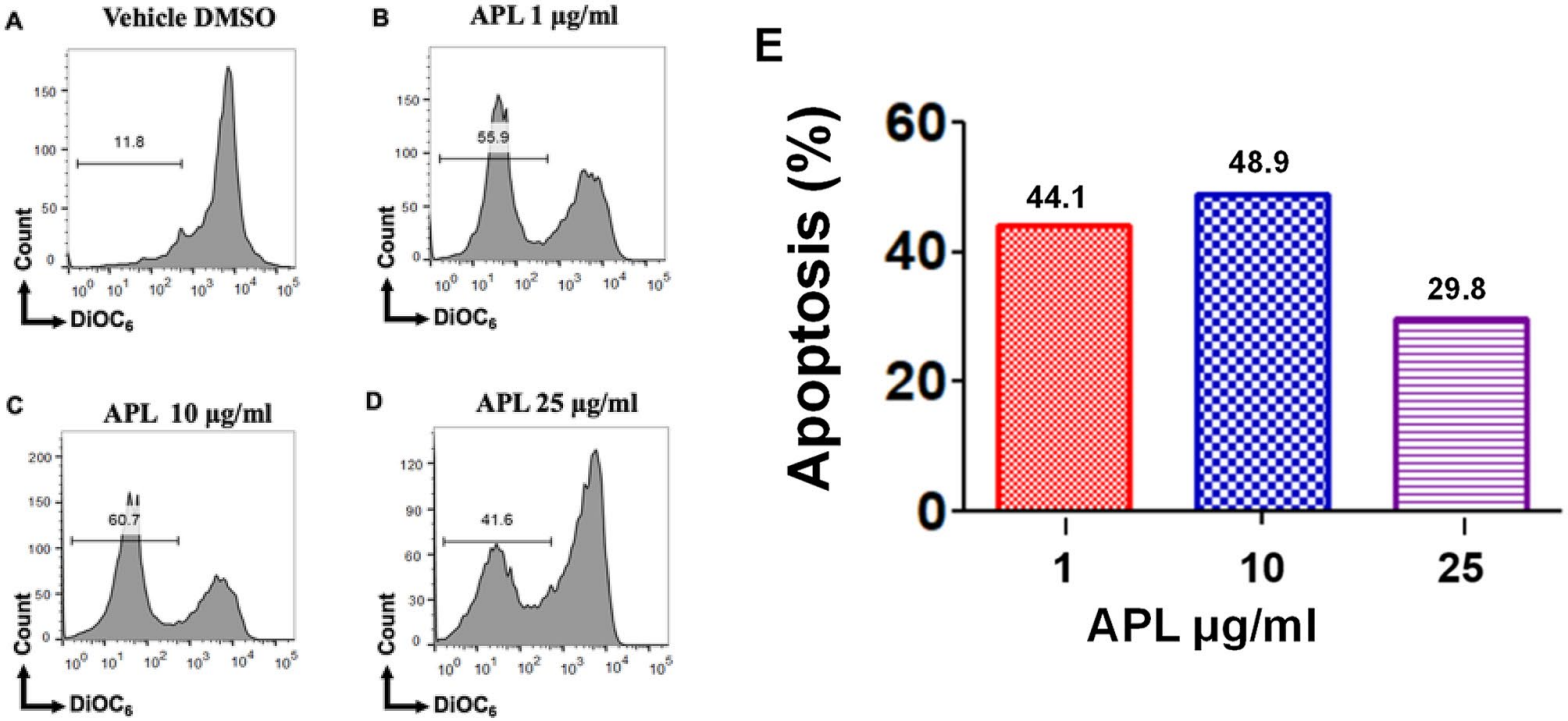
Effect of APL treatment on mitochondrial membrane potential (MMP) in B16 melanoma cells: This was determined using 3,3′-Dihexyloxacarbocyanine Iodide (DiOC6) after treatment with APL (*Psiadia lithospermifolia*) or vehicle (DMSO). (**A**) (vehicle DMSO), (**B**) (1 µg/ml), (**C**) (10 µg/ml), (**D**) (25 µg/ml) of APL extract. The data presented are representative of three independent experiments. (**E**) shows data as mean % apoptosis relative to vehicle controls from two independent experiments.

To test the role of the mitochondrial pathway in APL-induced apoptosis in EL4- and B16 cells, we examined loss of mitochondrial membrane potential (MMP) using DIOC_6_ dye (Figs. 8, 9). These data suggested that APL caused significant loss in MMP in both EL-4 and B16 cells although this was not dose-dependent. These data suggested that APL was inducing, at least in part, apoptosis through intrinsic pathway.

### Effect of plant extracts on Con-A activated splenocytes

**Figure 10:**
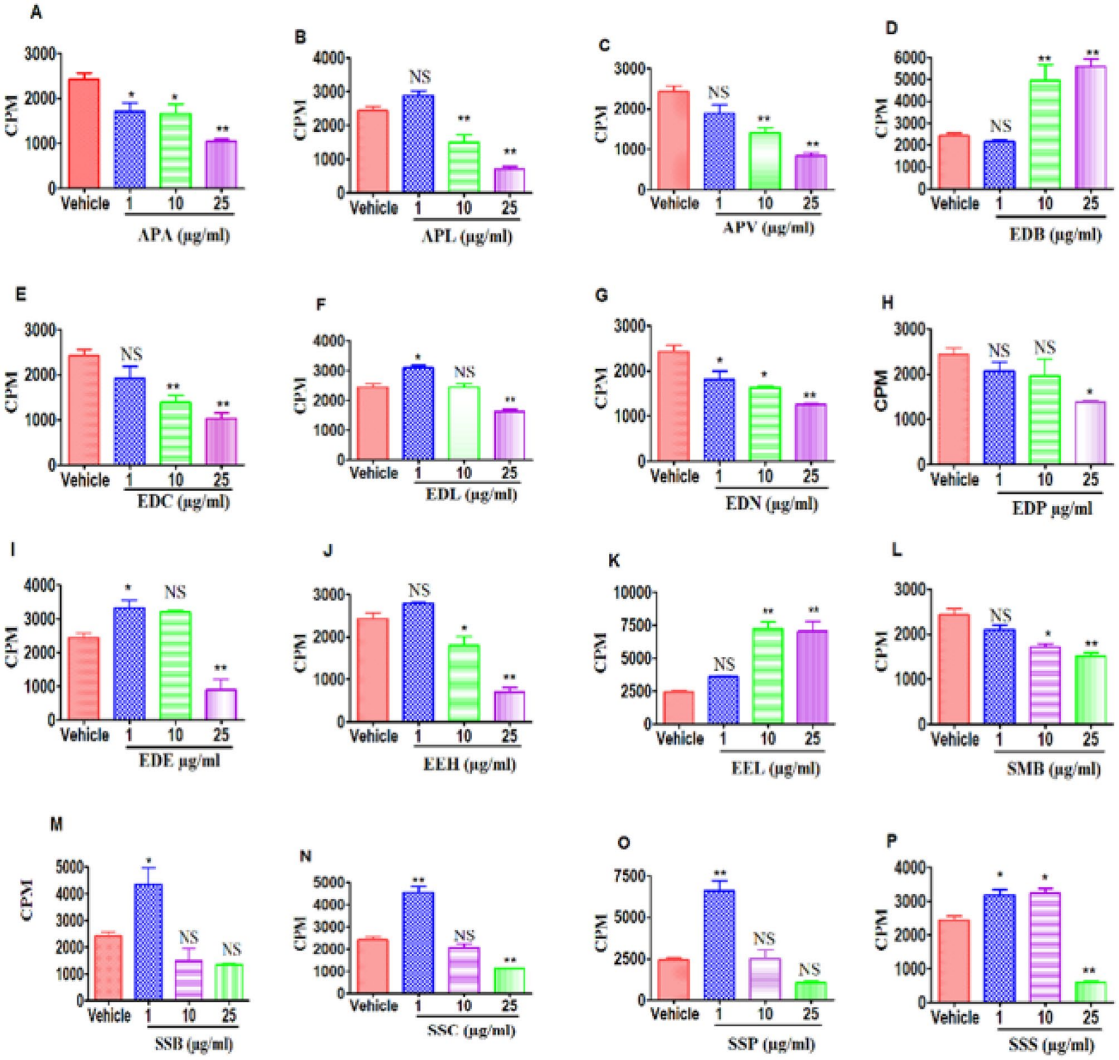
Effect of APL treatment on the proliferation of T cells: This was determined by ^3^H-Thymidine incorporation after 24 h treatment with plant extracts or vehicle, DMSO. (**A**–**C**) represent the Asteraceae family ((**A**) APA, (**B**) APL, (**C**) APV). (**D**–**I**) represent the Ebenaceae family ((**D**) EDB, (**E**) EDC, (**F**) EDL, (**G**) EDN, (**H**) EDP, (**I**) EDE). (**J**,**K**) represent the Erythroxylaceae family ((**J**) EEH, (**K**) EEL). (**L**–**P**) represent the Sapotaceae family ((**L**) SMB, (**M**) SSB, (**N**) SSC, (**O**) SSP, (**P**) SSS). The vertical bars represent mean ± S.E.M. of triplicate cultures and data were expressed as counts per minute (CPM). The significance between various groups was tested using one-way ANOVA where NS: Not significant; (*)p < 0.05, (**)p < 0.01, (***)p < 0.001.

To test if the Mauritius plant extracts can suppress inflammation, we investigated the effect of extracts on the proliferation of T cells. To that end, we activated spleen cells with T cell mitogen, Concanavalin A (Con-A). The results showed that extracts APA (Fig. 10A), APL (Fig. 10B) and APV (Fig. 10C) from Asteraceae, EDC (Fig. 10E) and EDN (Fig. 10G) from Ebenaceae, EEH (Fig. 10J) from Erythroxylaceae as well as SMB (Fig. 10L) from Sapotaceae decreased ConA-induced T cell proliferation at the higher concentrations of 10 and 25 μg/ml of dry crude leaf extract tested. Interestingly, EDB (Fig. 10D) from Ebenaceae and EEL (Fig. 10K) from Erythroxylaceae showed a dose-dependent increase in proliferative response. The extracts SSB (Fig. 10 M), SSC (Fig. 10N), SSP (Fig. 10O) and SSS (Fig. 10P) from Sapotaceae exhibited a biphasic response wherein at low doses, they enhanced the ConA-induced T cell proliferation but at higher concentrations decreased it.

### Effect of plant extracts on B cells

**Figure 11:**
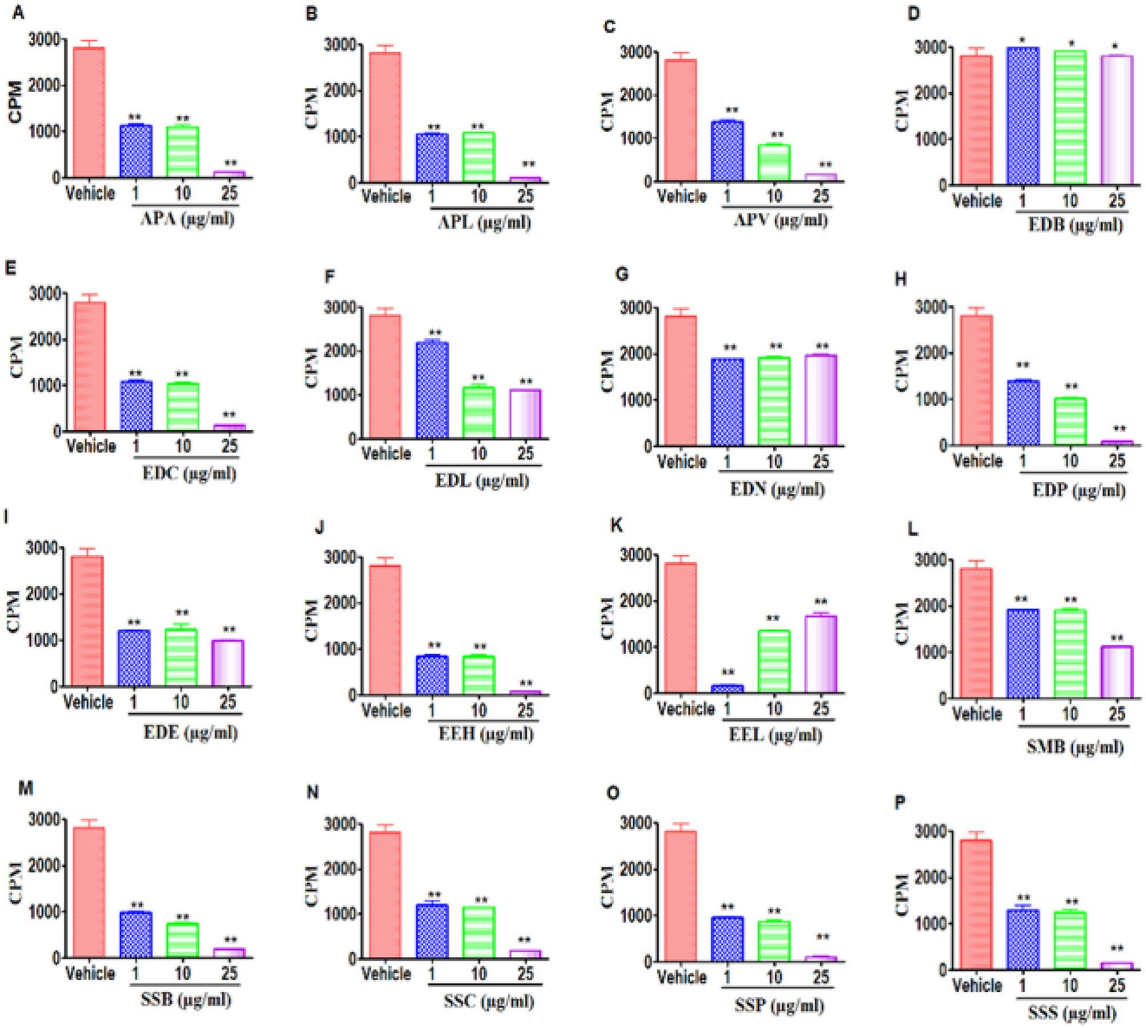
Effect of APL treatment on Proliferation of B cells: This was determined by ^3^H-Thymidine incorporation after 24 h treatment with plant extracts or vehicle, DMSO. (**A**–**C**) represent the Asteraceae family ((**A**) APA, (**B**) APL, (**C**) APV). (**D**–**I**) represent the Ebenaceae family ((**D**) EDB, (**E**) EDC, (**F**) EDL, (**G**) EDN, (**H**) EDP, (**I**) EDE). (**J**,**K**) represent the Erythroxylaceae family ((**J**) EEH, (**K**) EEL). (**L**–**P**) represent the Sapotaceae family ((**L**) SMB, (**M**) SSB, (**N**) SSC, (**O**) SSP, (**P**) SSS). The vertical bars represent mean ± S.E.M. of triplicate cultures and data were expressed as counts per minute (CPM). The significance between various groups was tested using one-way ANOVA where NS: Not significant; (*)p < 0.05, (**)p < 0.01, (***)p < 0.001.

We also determined the effects of Mauritian plant extracts on the proliferation of splenic B cells using a B cell mitogen, LPS (Fig. 11). The data showed that extracts APA (Fig. 11A), APL (Fig. 11B) and APV (Fig. 11C) from the Asteraceae, EDC (Fig. 11E) from Ebenaceae, EEH (Fig. 11J) from Erythroxylaceae and SSB (Fig. 11M), SSC (Fig. 11N), SSP (Fig. 11O) and SSS (Fig. 11P) from Sapotaceae significantly decreased the LPS-induced B cell proliferation at higher concentration of 25 μg/ml of dry crude leaf extract. However, extracts SMB (Fig. 11L) from Sapotaceae and EDL (Fig. 11F) from Ebenaceae showed biphasic response wherein at low doses, they enhanced B cell proliferation. However, extracts EEL (Fig. 11K) showed dose-dependent increase in proliferative response. The extracts of EDB (Fig. 11D), EDN (Fig. 11G) did not inhibit B cell proliferation at 25 µg/ml of dry crude leaf extract.

### Phytochemical screening of Mauritius endemic plants extracts

**Figure 12:**
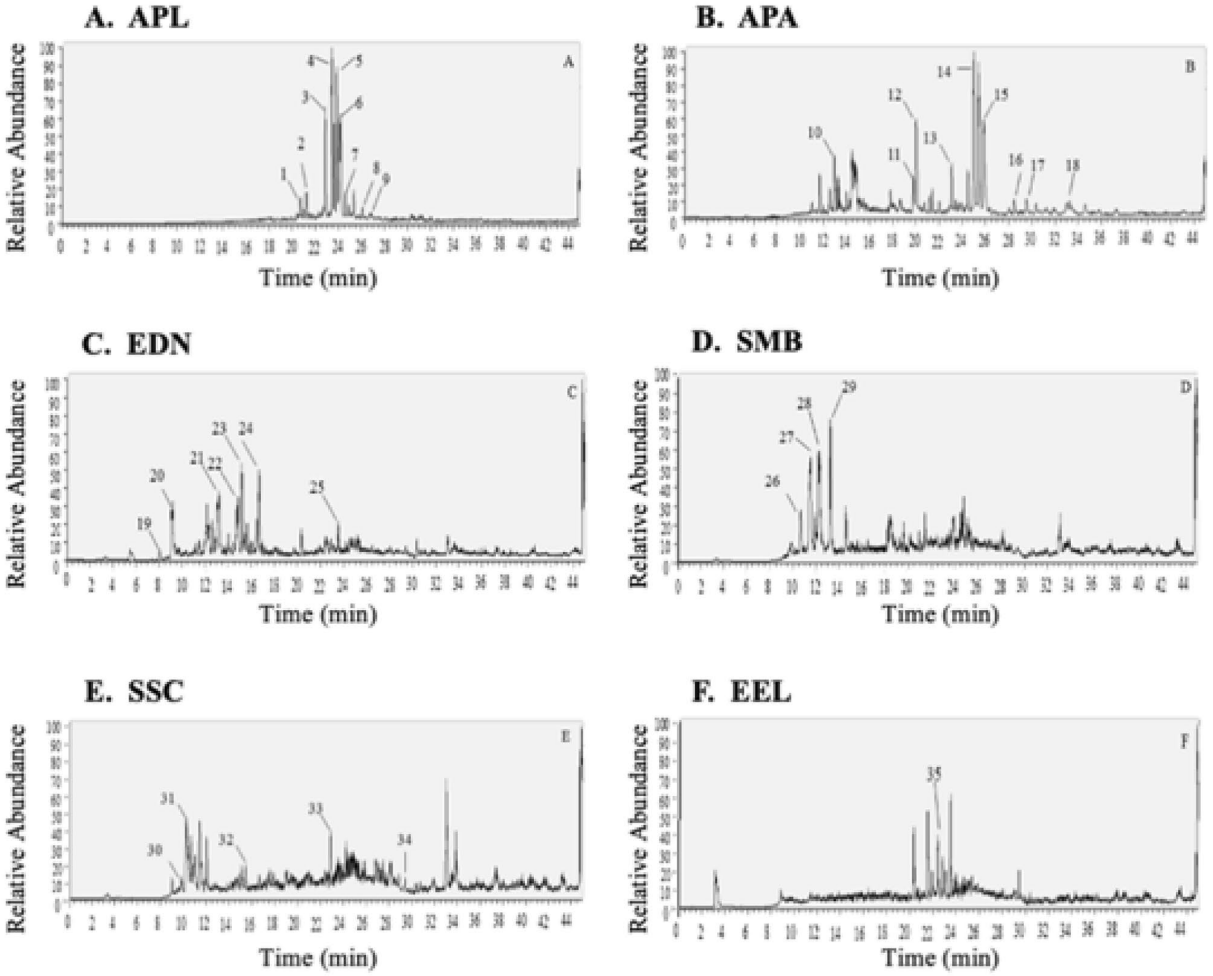
Preliminary phytochemical screening of Mauritius selected endemic plants: This was determined using LC–MS chromatography in positive mode. (**A**) represents plant extract of *Psiadia lithospermifolia*, (**B**) represents plant extract of *Psiadia arguta*, (**C**) represents plant extract of *Diospyros nodosa* (**D**) *Mimusops balata* (**E**) *Sideroxylon cinereum* and (**F**) *Erythroxylum laurifolium*.

**Figure 13:**
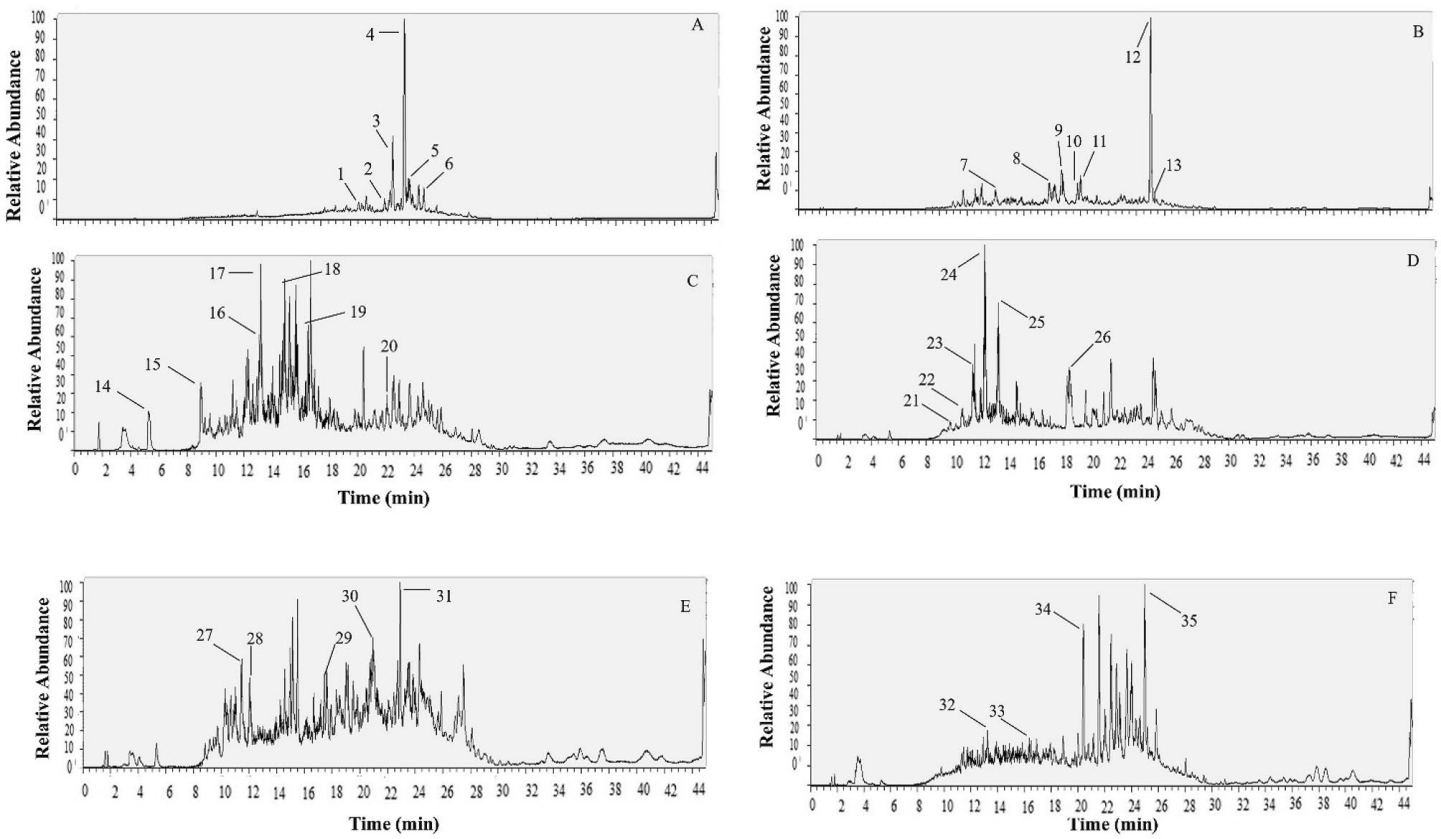
Preliminary Phytochemical screening of Mauritius selected endemic plants: This was determined using LC–MS chromatography in negative mode. (**A**) represents plant extracts from: *Psiadia lithospermifolia*, (**B**) *Psiadia arguta,* (**C**) *Diospyros nodosa,* (**D**) *Mimusops balata* (**E**) *Sideroxylon cinereum* and (**F**) *Erythroxylum laurifolium.*

Six plant species namely *Psiadia arguta*, *Psiadia lithospermifolia*, *Diospyros nodosa, Sideroxylon cinereum, Mimusops balata*, and *Erythroxylum laurifolium* were selected for phytochemical screening using high mass resolution LC–MS analysis, with tentative detection of the main class of secondary metabolites presented in Figs. 12 and 13. Peak number, retention time, observed protonated molecular formula, and protonated M/Z value for individual components are presented in Tables 2 and 3. Metabolite assignments were made by comparing the detected compounds MS data in positive and negative mode with cited literature. Identified metabolites (Tables 2, 3) were mainly phenols, flavonoids, terpenes and terpenoids, and fatty acids. The elution order of the chromatographic peaks correlated with decreasing polarity, whereby polar compounds were eluted first followed by semi-polar and finally nonpolar compounds.

**Table 2:** Phytochemicals identified in methanolic extracts of endemic plants species after LC–MS in positive ionization mode.

Peak no.*	Rt (min)	Protonated [M + H] ^+^M/Z value	Protonated molecular formula	Secondary metabolites
***Psiadia lithospermifolia***				
1	20.74	317.21204	C20H29O3	Cafestol
2	21.25	277.21777	C18H29O2	Stearidonic acid
3	22.85	321.24268	C20H33O3	Isocupressic acid
5	23.85	287.23779	C20 H31 O	Taxadienone
4	23.43	307.26385	C20H35O2	Incensole
6	24.20	291.26880	C20 H35 O	Geranylgeraniol
7	24.91	305.24829	C20H33O2	Unidentified
8	26.10	303.23285	C20 H31 O2	Levopimaric acid
9	26.73	335.25845	C21 H35 O3	Unidentified
***Psiadia arguta***				
10	19.81	375.10925	C19 H19 O8	Methyoxylated flavonol
11	20.01	405.11993	C20 H21 O9	O-Methylated flavonol
12	23.10	449.14548	C22 H25 O10	Sakuranin
13	25.02	307.26349	C20 H35 O2	Incensole
14	25.87	293.28467	C20 H37 O	Diterpene alcohol
15	28.47	333.27951	C22 H37 O2	Fatty acid
16	29.53	335.29468	C22 H39 O2	Fatty acid
17	33.27	377.34232	C25 H45 O2	Unidentified
18	34.63	391.35834	C26 H47 O2	Unidentified
***Diospyros nodosa***				
19	8.07	315.07159	C13 H15 O9	Norbergenin
20	9.12	329.08716	C14 H17 O9	Bergenin
21	13.19	287.05576	C15 H11 O6	Kaempferol
22	14.87	445.11441	C15 H25 O15	Unidentified
23	15.18	433.11411	C21 H21 O10	Kaempferol 7-rhamnoside
25	16.67	459.13019	C23 H23 O10	Unidentified
***Mimusops balata***				
26	10.68	319.04568	C15 H11 O8	Flavonols
27	11.52	333.06100	C16 H13 O8	Myricetin
28	12.22	303.05026	C15 H11 O7	Quercetin
29	13.24	317.06573	C16 H13 O7	O-methylated flavonol
***Sideroxylon cinereum***				
30	9.27	307.08249	C15 H15 O7	Epigallocatechin
31	10.26	303.05029	C15 H11 O7	Quercetin
32	15.47	455.35364	C30 H47 O3	Unidentified
33	22.89	487.34332	C30 H47 O5	Unidentified
34	29.14	439.35742	C30 H47 O2	Unidentified
***Erythroxylum laurifolium***				
35	22.43	355.15997	C14 H27 O10	Unidentified

*Peak numbers are represented on LC–MS chromatograms (positive mode) in Fig. 12.

**Table 3:** Phytochemicals identified in methanolic extracts of endemic plants species after LC–MS in negative ionization mode.

*Peak no	Rt (min)	Deprotonated [M − H]^−^ M/Z value	Deprotonated molecular formula	Secondary metabolites
***Psiadia lithospermifolia***				
1	21.6	485.31479	C26H45O8	Unidentified
2	22.32	485.31381	C26H46O8	Diterpene
3	22.87	293.21317	C18H29O3	Fatty acid
4	23.6	321.24426	C20H33O3	Diterpene
5	23.95	295.22873	C18H32O3	Fatty acid
6	24.97	471.35013	C30H47O4	Triterpenes
***Psiadia arguta***				
7	14.64	515.12268	C25 H23O12	Hydroxycinnamic acid
8	17.79	419.10049	C13 H23 O15	Unidentified
9	18.67	567.28558	C29H43O11	Glycosides
10	19.81	373.09540	C12 H22 O13	Unidentified
11	20.04	403.10489	C20 H19O9	Flavonols
12	25.0	323.25977	C20H35O3	Labdanolic acid
13	25.27	355.28690	C21H39O4	Oleic acid
***Diospyros nodosa***				
14	5.24	345.08362	C14H17O10	Bergenin hydrate
15	8.93	327.07324	C14 H15 O9	Bergenin
16	13.06	421.11588	C20 H21 O10	Carboxylic acid
17	13.18	417.08453	C20 H17 O10	Flavonols
18	14.72	447.13007	C22 H23 O10	Flavanone glucosides
19	15.95	439.11023	C16 H23 O14	Carboxylic acid
20	22.05	487.34674	C30 H47 O5	Pentacyclic triterpenoid
***Mimusops balata***				
21	9.76	577.13873	C30 H25 O12	Proanthocyanidins
22	10.66	479.08511	C21 H19 O13	Flavonoid glycosides
23	11.40	463.08920	C21 H19 O12	Glycosyloxyflavone
24	12.24	477.10410	C22 H21 O12	Flavonoid-7-o-glycosides
25	13.23	461.10907	C22 H21 O11	Isoflavone
26	18.43	503.33975	C30 H47 O6	Pentacyclic triterpenoid
***Sideroxylon cinereum***				
27	11.48	463.09064	C21 H19 O12	Unidentified
28	12.05	433.07877	C20 H17 O11	Quercetin O-glycoside
29	17.60	501.32443	C30 H45 O6	Triterpenoids
30	20.96	487.34515	C30 H47 O5	Pentacyclic triterpenoid
31	22.96	293.21317	C18 H29 O3	Lineolic acids
***Erythroxylum laurifolium***				
32	13.24	543.13202	C30 H23 O10	Proanthocyanidin
33	16.35	329.23541	C18 H33 O5	Fatty acids
34	24.0	295.22910	C18 H31 O3	Linoleic acid
35	24.99	323.26004	C20 H35 O3	Fatty acid

*Peak numbers are represented on LC–MS chromatograms (positive mode) in Fig. 13.

The chromatogram of *Psiadia lithospermifolia* in positive mode (Fig. 12A) was characterized by one main region eluting at 20.74 to 26.73 min and in negative mode (Fig. 13A), one main region eluting at 21.6 to 24.97 min with peaks showcasing the occurrence of diterpenes, fatty acids, diterpene acid, and diterpene alcohol. However, the chromatogram of *Psiadia arguta* shown in positive mode Fig. 12B displayed peaks in two regions: the first half regions (19.81 to 23.10 min) showed polar compounds tentatively identified as flavonoids and the second eluted in the region (25.02 to 34.63 min) indicated diterpenes, alcohol and fatty acid. However, in negative mode, peaks eluted in the region (14.64 to 25.27). Figure 13B indicated the presence of phenolics and fatty acids. In positive mode (Fig. 12C), main peaks at 8.07 to 15.18 min, and in negative mode peaks at 5.24 to 15.95 min (Fig. 13C) for *Diospyros nodosa* from Ebenaceae were detected mainly as polyphenolic, phenolic acid and flavonoids, whereas the peak at 22.05 min (Fig. 13C) min showed a pentacyclic triterpenoid. Both the positive and negative mode of the chromatogram of *Mimusops balata* (Figs. 12D, 13D) was dominated in polyphenolic compounds (10.68–13.24 min) (Table 2) and (9.76 to 13.23 min) (Table 3). The peak at 18.43 min in negative mode (Fig. 13D) showed the occurrence of Pentacyclic triterpenoid. On the other hand, the chromatogram of *Sideroxylon cinereum* in positive mode (Fig. 12E) was categorized with peaks (9.27–10.26 min) representing flavonoids, and peaks in the region (15.47–22.89 min) were identified as triterpene. Peaks displayed in the negative mode (Fig. 13E) (11.48 to 22.96) were mostly flavonoids, terpenoids, and fatty acid. We were unable to identify the only peak (22.43) from *Erythroxylum laurifolium* in positive mode of the chromatogram (Fig. 12F). However, in negative mode (Fig. 13F) polyphenols and fatty acid were detected in the region (13.24 to 24.99).

## Discussion

In the current investigation, the anti-cancer and anti-inflammatory properties of selected Mauritius endemic plant families including Asteraceae, Ebenaceae, Erythroxylaceae, and Sapotaceae traditionally used in the Mauritius folklore as medicines were studied. We also performed a preliminary LC–MS screening to detect the presence of major phytoconstituents from these plants. To study the anti-cancer properties, we investigated the effect of plant extracts to mediate apoptosis in EL-4 lymphoma and B16 melanoma cells. Additionally, to study the anti-inflammatory properties, we tested the effect of the plant extracts on the proliferation of T and B cells. In the current study, we found that a significant number of plant extracts exhibited both anti-cancer and anti-inflammatory effects. Interestingly, some extracts were also able to enhance the proliferation of T and B cells thereby suggesting that such extracts exhibit the properties of immunopotentiators.

It was evident from our MTT cytotoxic results that extracts of Asteraceae plants (APA, APV, and APL) at higher doses 25 µg/ml of dry crude leaf extract significantly reduced viability of EL4 and B16 cells compared to other plant families as shown in (Figs. 2A–C, 3A–C). APL turned out to be more cytotoxic than other extracts thereby making it the best candidate for further apoptotic study. Other plant species including Ebenaceae (EDC, EDL and EDB), Erythroxylaceae (EEH and EEL), and Sapotaceae (SMB, SSB, SSC and SSS) were also effective at inhibiting the growth of B16 melanoma cells but not EL-4 cells. Although Mauritius endemic plants are commonly used in the management of health conditions on the island, studies on their mechanisms of anti-cancer and anti-inflammatory efficacy and action are extremely limited. So far, Mauritius endemic plant families Euphorbiaceae (*Acalypha integrifolia* Willd) and Myrtaceae (*Eugenia tinifolia* Lam) have been screened for their anti-cancer properties; they were shown to be cytotoxic against human oesophageal squamous cell carcinoma (KYSE-30) at IC50 of 6.42 ± 2.21 and 6.99 ± 0.38 0 µg LW/ml of extracts respectively^14^. In addition, there is only one study which reported that *Psiadia arguta* endemic to Mauritius Island and *Psiadia dentata* (Cass.) DC endemic to Reunion Island, display anti-proliferative effects on human colorectal adenocarcinoma (DLD-1) and human lung carcinoma (A549)^15,16^. However, both plant species turned out to be cytotoxic with IC50 of 24 and 43 µg/ml respectively on human normal fibroblasts. Also, the anti-proliferative properties of *Psiadia punctulata* (DC.) Vatke. originally from East Africa have also been studied.

In the current study, we confirmed the apoptotic activity of APL through additional tests such as annexin/PI assay. Our data showed that APL induced early and late apoptosis ultimately leading to necrosis in a time and dose-dependent fashion compared to vehicle-treated controls (Figs. 4, 5). These data suggested that the reductions in the number of viable cells mediated by APL result from induction of apoptosis.

We investigated the mechanisms of apoptosis induced by the methanol extract of APL on EL4 and B16 melanoma cell lines by studying both mitochondrial (intrinsic) and death receptor (extrinsic) pathways. Both pathways are caspase-dependent pathways. In extrinsic pathways, stimulation of external factors promotes the interaction between death receptor (Fas) and Fas ligand (FasL) leading to activation of Caspase 8, which in turn activates the apoptosis executioner, Caspase 3^17^. In intrinsic pathways, the mitochondrial outer membrane is permeabilized in response to intracellular stress leading to the loss of Mitochondrial Membrane Potential and opening of the permeability transition pore. Subsequently, cytochrome C is released from the mitochondria to the cytoplasm which triggers a series of events that activated the apoptotic initiator caspase 9 leading to activation of apoptosis executioner, Caspase 3^18^. In the current study, upon blocking Caspase 8 in EL4 cells treated with methanol extracts of APL, we noted significant blocking of apoptosis (Fig. 6H). A similar trend was noted in APL-induced apoptosis in B16 cells (Fig. 7H). However, when EL4 and B16 cells were treated with APL, decreased MMP was also noted (Figs. 8, 9), explained by the fact that the apoptosis-inducing compounds found in APL may trigger both pathways of apoptosis, similar to resveratrol that we have reported previously^12^. Alternatively, the APL, being a crude extract, may have multiple apoptosis-inducing compounds as revealed by the LC–MS data, each using only one of the pathways and thus, in combination, promote both pathways of apoptosis. Synergistic anticancer effects are highly appraised for efficient induction of cancer cell death in combination with therapy and delayed drug resistance.

Many anti-cancer agents such as cyclophosphamide have also been shown to kill rapidly proliferating cells, such as activated immune cells, thereby acting as anti-inflammatory agents^19^. Moreover, some cancers are driven by chronic inflammation, and thus, suppressing inflammation has a beneficial effect in preventing the development of cancers. To that end, in the current study, we also investigated the effect of Mauritius endemic plant extracts on T and B cells that were activated with specific mitogens such as Con-A and LPS, respectively (Figs. 10, 11). T and B lymphocytes are the major components of the adaptive immune response. Our results showed that at a high concentration of 25 µg/ml of dry crude leaf extract, most plant species from Asteraceae (APA, APL, and APV), Ebenaceae (EDC, EDL, EDN, EDE, and EDP), and Erythroxylaceae (EEH), and Sapotaceae (SSC, SSP, SSS, and SMB) inhibited the proliferation of Con-A stimulated T cells and LPS-activated B cells. It was interesting to note that most of the plant species which displayed apoptotic action against EL4 or B16 melanoma cells also inhibited the proliferation of T and B cells, previous studies showed that methanol extracts of *Psiadia arguta* (APA) and *Psiadia dentata* (Asteraceae) mediated inhibitory activity against Raw 264.7 murine macrophages activated by LPS16. We also noted that some plant extracts from Ebenaceae (EDB) and Erythroxylaceae (EEL) enhanced T cell proliferation in a dose-dependent manner while not affecting B cell proliferation. Plant products have been previously shown to mediate immuno-stimulatory properties. For example, extracts from *Tinospora crispa* (L.) Hook. f. & Thomson*, *a tree that belongs to the family Menispermaceae was shown to enhance T cell proliferation induced by ConA^20^. Overall, in the current work, we were able to identify several immunomodulatory properties of extracts from plant families Ebenaceae, Sapotaceae, and Erythroxylaceae. To our knowledge, no studies have been carried out previously to investigate the anti-inflammatory and immunomodulatory properties of these plant species, and thus, our studies form the basis for further investigations on identifying and isolating bioactive molecules and studying their in vivo activities.

As a preliminary phytoconstituent screening, we detected the presence of major phytochemicals from selected *Psiadia* plants using LC–MS analysis and it is not surprising that we detected the presence of terpenes and fatty acids as major components in APL (*Psiadia lithospermifolia*) (Tables 2, 3). The genus *Psiadia* from the Mascarene Island is well reputed for the naturally occurring essential oils, which are made up mainly of monoterpenes, sesquiterpenes, aliphatics, and other shikimic acid derivatives but little is mentioned on their potential anti-cancer activity^1,8,21,22^. The presence of cafestol a diterpenoid has previously been shown to induce apoptosis in MSTO-211H^23^. The observed occurrence of geranylgeraniol from APL suggests its role in the inhibitory effects on the proliferation of mitogen-activated T and B cells. Indeed, geranylgeraniol effectively suppressed inflammation in Mevalonate kinase deficiency MKD mouse model^24^. Further, we noted that phenolic compounds predominate in APA, EDN, SMB, and SSC (Table 2). Phenolic compounds are highly appraised for their chemotherapy, chemopreventive, anti-inflammation, and immunomodulation properties. It was no surprise to detect the presence of phenolic compounds among the studied endemic plants. Previous studies have reported the polyphenolic richness of the Mauritius endemic flora^4,22^. Thus, the cytotoxic activities of APA, SMB, and SSC may be attributed to the presence of various phenolic compounds detected. We noted the occurrence of O-methylated flavonol, a flavonoid from APA which has been shown to significantly reduce inflammation in rat paw edema^25^. Interestingly, the LC–MS profile of EDN showed the occurrence of bergenin compounds, which have been shown to inhibit inflammation via the modulation of MAPK and NF-kB signaling pathways in a mouse model of LPS-induced mastitis^26^. On the other hand, another study^27^, showed bergenin to induce Th1 immune responses in a murine model of *Mycobacterium tuberculosis* infection.

## Conclusion

In summary, our study provides important evidence on the potential anti-cancer, anti-inflammatory and immunopotentiating properties of Mauritius endemic plants, further validating and supporting their traditional use. This study forms the basis of future work to isolate, characterize and elucidate important phytochemicals, which will not only be relevant for their therapeutic potential but will also sensitize relevant authorities on the importance of conserving the Mauritius endemic floral biodiversity.

## Abbreviations

EL4: T cell lymphoma
B16: B16F10 Melanoma cell
T cell: T lymphocyte
B cell: B lymphocyte
MMP: Mitochondrial membrane potential
LC–MS: Liquid chromatography–mass spectrometry
MTT: 3-(4,5-Dimethyl-2-thiazolyl)-2,5-diphenyl-2H-terazolium bromide
DiOC_6_: 3,3′-Dihexyloxacarbocyanine Iodide
